# ON THE THRESHOLD: CREATIVITY IN MID-LIFE

**DOI:** 10.1093/geroni/igac059.843

**Published:** 2022-12-20

**Authors:** Carolyn Adams-Price, Desmond O'Neill

## Abstract

While late-life creativity has attracted significant scholarly inquiry, it is not clear that the same focus has applied to creativity in midlife and its relationship to creativity across the lifespan. Allied to a proposed symphonic concert by the Indianapolis Symphony of works from late midlife creativity with gerontologically-themed pre-concert talks occurring alongside GSA 2022, an activity curated by the Humanities, Arts and Cultural Gerontology Advisory Panel of GSA, this symposium will provide insights into a relatively under-explored aspect of creativity and aging, that of midlife creativity and its relationship to creativity at other stages of life. For many artists the most significant breakthroughs in style and critical reception are found in the works of midlife, and the symposium will explore the phenomenology and underlying themes of midlife creativity. This will include a review of the literature, a focus on midlife breakthrough and development in the creativity of Brahms and Janacek, interaction with the program of the parallel symphony concert, and a focus on arts and crafts creativity in people newly undertaking arts and crafts in mid-life. This will be complemented by a review of creativity and well-being from the age of 50 onwards.

